# DGKα, Bridging Membrane Shape Changes with Specific Molecular Species of DAG/PA: Implications in Cancer and Immunosurveillance

**DOI:** 10.3390/cancers14215259

**Published:** 2022-10-26

**Authors:** José Carlos Bozelli, Richard M. Epand

**Affiliations:** Department of Biochemistry and Biomedical Sciences, McMaster University, Health Sciences Centre, Hamilton, ON L8S 4K1, Canada

**Keywords:** cancer, immune surveillance, immunotherapy, diacylglycerol kinase α, diacylglycerol, phosphatidic acid, substrate acyl chain specificity, regulation of enzymes by membrane shape, cellular signaling

## Abstract

**Simple Summary:**

DGKα is being considered as a target in immunotherapy. An advantage of targeting this enzyme is that it potentially has a dual role, i.e., it would both decrease the proliferation of cancer cells and incite the immunological response of T-lymphocytes. Here, the modulation of DGKα enzymatic activity and substrate acyl chain specificity by membrane shape and its potential implications in cancer and immune cell biology are reviewed.

**Abstract:**

Cancer immunotherapy has revolutionized the oncology field. Despite the success, new molecular targets are needed to increase the percentage of patients that benefits from this therapy. Diacylglycerol kinase α (DGKα) has gathered great attention as a potential molecular target in immunotherapy because of its role in cancer proliferation and immunosuppression. DGKα catalyzes the ATP-dependent phosphorylation of diacylglycerol (DAG) to produce phosphatidic acid (PA). Since both lipids are potent signaling messengers, DGKα acts as a switch between different signaling pathways. Its role in cancer and immunosuppression has long been ascribed to the regulation of DAG/PA levels. However, this paradigm has been challenged with the identification of DGKα substrate acyl chain specificity, which suggests its role in signaling could be specific to DAG/PA molecular species. In several biological processes where DGKα plays a role, large membrane morphological changes take place. DGKα substrate specificity depends on the shape of the membrane that the enzyme binds to. Hence, DGKα can act as a bridge between large membrane morphological changes and the regulation of specific molecular species of DAG/PA. Bearing in mind the potential therapeutic benefits of targeting DGKα, here, the role of DGKα in cancer and T cell biology with a focus on the modulation of its enzymatic properties by membrane shape is reviewed. The goal is to contribute to a global understanding of the molecular mechanisms governing DGKα biology. This will pave the way for future experimentation and, consequently, the design of better, more potent therapeutic strategies aiming at improving the health outcomes of cancer patients.

## 1. Introduction

Cancer continues to be one of the major health problems globally, bringing extreme physical, emotional, and financial strain on individuals, communities, and healthcare systems. However, in the past ten years, there has been a tremendous improvement in the survival and quality of life of cancer patients, especially terminal ones. This was brought by a change in the landscape of cancer treatment from previous standard of care (e.g., chemo- and radiotherapy) to the new era of immunotherapy [1,2,3]. In cancer immunotherapy, the idea is to resume the ability of the immune system, e.g., T-cells, to recognize and fight tumor cells. Cancer immunotherapy has revolutionized the oncology field and it has now established itself as a pillar of cancer treatment. One of the strategies in cancer immunotherapy is the immune checkpoint blockade, which relies on blocking negative regulators of T cells and, therefore, incites powerful T cell responses [4,5,6]. Immune checkpoint inhibitors often target receptors on the surface of immune cells, which in a tumor microenvironment are acting to silence the activation of T cells. Despite the increased success of immune checkpoint blockade against a range of cancers, currently only a small percentage of patients show significant improvement in health outcomes. Hence, currently, there is a need to find new molecular targets aiming at improving efficacy and broaden the use of immune checkpoint blockade.

Recently, there has been an increased interest in diacylglycerol kinase α (DGKα), an intracellular lipid kinase, as a potential target in immune checkpoint blockade [7,8,9]. This is because the silencing of DGKα activity provides a two-pronged attack on cancer cells. On the one hand, DGKα has been found to increase the survival, proliferation, migration, and invasion of some cancers and, therefore, its inhibition has been shown to prevent tumorigenesis [9,10,11,12,13,14]. On the other hand, DGKα has been shown to decrease activation and increase anergy, a hypo-responsive state, in T cells and, therefore, its inhibition has been shown to incite powerful T cell responses leading to greater immune clearance of cancer cells [15,16,17,18]. DGKα is an interfacial enzyme that utilizes ATP to phosphorylate diacylglycerol (DAG) at the membrane-water interface, yielding phosphatidic acid (PA). Both DAG and PA are potent lipid signaling molecules. It has been proposed that the DGKα role in cancer and immune cell biology is due to its ability to regulate the levels of these two lipid messengers. However, an idea that started to emerge is that it is not only the levels of DAG and PA, but also the nature of their molecular species that plays a role in the biological processes controlled by DGKα. This is based on the finding that DGKα exhibits substrate acyl chain specificity for its DAG substrate, a property that has been shown to be modulated by the shape of the membrane the enzymes bind to [19,20,21,22,23]. Bearing in mind the potential therapeutic benefits of targeting DGKα, it is here discussed the role of DGKα in cancer and T cell biology. However, contrary to other reviews on the topic, here the focus of the discussion will be on the modulation of DGKα enzymatic activity and substrate acyl chain specificity by the membrane shape and its potential implications in cancer and immune cell biology. By focusing on this other aspect of DGKα biology, the goal is to contribute to build a global understanding of the molecular mechanisms governing these biological processes. This will pave the way for further experimentation and, consequently, the design of better and more potent therapeutic strategies in immunotherapy with the goal of improving the health outcomes of cancer patients.

## 2. DGKα

### 2.1. Enzymatic Reaction and Substrate Acyl Chain Specificity

**Figure 1:**
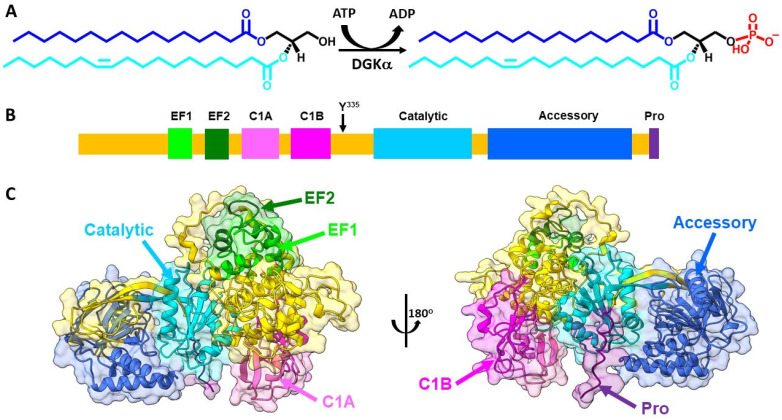
DGKα enzymatic reaction and structure. (**A**) DGKα catalyzes the ATP-dependent phosphorylation of diacylglycerol (DAG) to produce phosphatidic acid (PA). DAG and PA molecular species bear 16:0 (blue)/18:1 (cyan) acyl chains. This DAG molecular species illustrates an example of DGKα preferred substrate. (**B**) Domain architecture of DGKα includes two EF hand motifs (EF hand 1 in lime and EF hand 2 in forest green), two C1 domains (C1A in hot pink and C1B in magenta), a catalytic domain (dark turquoise), and an accessory domain (royal blue). Y^335^ (black arrow), which is involved in DGKα phosphorylation, is identified as well as a proline rich segment (Pro, purple), suggested to be involved in membrane interaction. (**C**) AlphaFold predicted high-resolution structure of human DGKα (UniprotKB accession number P23743) was obtained from AlphaFold database [25]. Structures were generated using Chimera X [26] and domains colored as follows: EF hand 1 (residues 114–142, lime), EF hand 2 (residues 159–187, forest green), C1A (residues 206–253, hot pink), C1B (residues 268–319, magenta), catalytic (residues 376–500, dark turquoise), accessory (residues 520–701, royal blue), and Pro (residues 722–735, purple). The remaining protein was colored in gold.

DGKα is a member of the family of lipid kinases namely diacylglycerol kinases (DGK). In humans, ten paralogues of DGK have been identified. The presence of gene splice variants increases the number of members of this family, highlighting the importance of the enzymatic reaction carried out by this family of enzymes. All DGK catalyze the same biochemical reaction, i.e., the ATP-dependent phosphorylation of DAG to produce PA (Figure 1A). However, the different expression profile, subcellular location, structure, regulatory motifs, and substrate specificity suggest that each DGK paralog bears different biological functions, albeit catalyzing the same biochemical reaction. While for a long time it was believed that DGKε was the only paralog with substrate acyl chain specificity, recently it was shown that DGKα also presents substrate acyl chain specificity [20,24]. In celulla, it has been shown that DGKα bears specificity for DAG molecular species containing saturated/monounsaturated acyl chains [19,21,22]. This same acyl chain specificity was corroborated by a systematic in vitro study with purified DGKα and model membranes of variable physicochemical properties [23].

### 2.2. Structural Properties

DGKα is an 80 kDa cytosolic DGK that in healthy humans has enhanced expression in esophagus, skin, and lymphoid tissue (www.proteinatlas.org (accessed on 9 August 2022)) [27]. DGKα is one of three type I DGK, which are characterized by bearing EF hand motifs. The domain architecture of DGKα involves the presence of two EF hand motifs in the N-terminus followed by two C1 domains, the catalytic domain, and an accessory domain close to the enzyme C-terminus (Figure 1B). The EF hand motifs are regulatory motifs that bind Ca^2+^, while the C1 motifs have been shown to mediate DAG acyl chain specificity [22,28]. The catalytic domain is a conserved domain of lipid kinases responsible for carrying out the enzymatic reaction and the accessory domain, believed to assist with catalysis. The tridimensional high-resolution structure of full length DGKα has not been solved experimentally yet. However, recent advancements in the prediction of the structure of proteins by artificial intelligence has shed some light on its structure [25,29]. The Alphafold predicted structure of DGKα yields a globular protein where most of its domains are alpha-helical folded and the accessory domain exhibit mixtures of alpha-helix and beta-sheet secondary structures (Figure 1C).

**Figure 2:**
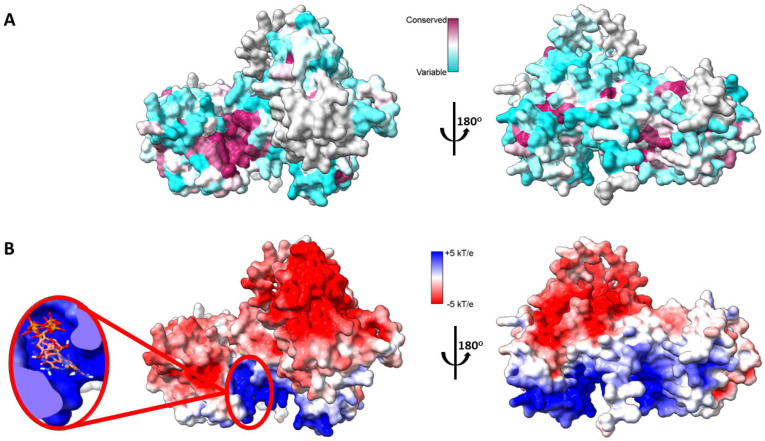
Mapping DGKα three-dimensional structure. (**A**) Sequence conservation of human DGK mapped onto AlphaFold predicted high-resolution structure of human DGKα. Multiple sequence alignment of the ten paralogs of human DGK was done with Clustal Omega [33] and residues were colored based on sequence conservation (maroon = conserved and cyan = variable residues). (**B**) Electrostatic surface potential (ESP) of AlphaFold predicted high-resolution structure of human DGKα. ESP was calculated with adaptive Poisson-Boltzman solver (APBS) [34] and colored according to color key (red = acidic, white = neutral, and blue = basic surfaces). Enlarged on the left panel ATP docked to its putative binding site, which binds with an average affinity of −7.3 ± 0.6 kcal.mol^−1^. The three lowest energy docking poses are shown. Blind docking was performed with Webina [35].

**Figure 3:**
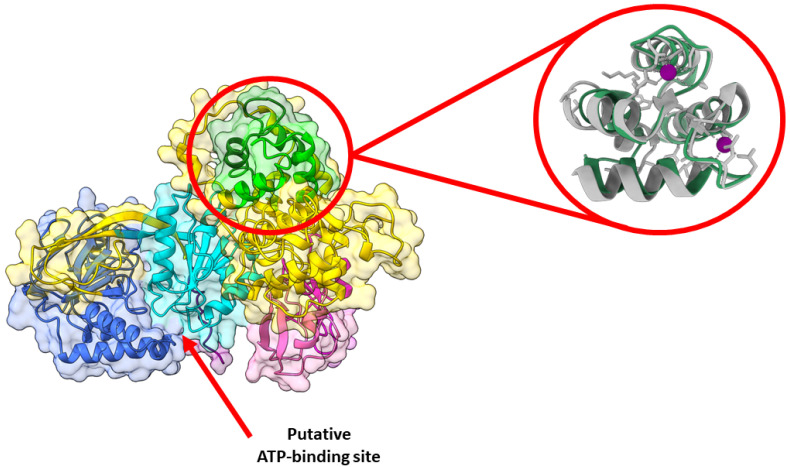
DGKα predicted structure is likely the active one. AlphaFold predicted high-resolution structure of human DGKα (UniprotKB accession number P23743). Structures were generated using Chimera X and domains colored as follows: EF hand 1 (residues 114–142, lime), EF hand 2 (residues 159–187, forest green), C1A (residues 206–253, hot pink), C1B (residues 268–319, magenta), catalytic (residues 376–500, dark turquoise), accessory (residues 520–701, royal blue), and Pro (residues 722–735, purple). The remaining protein was colored in gold. Enlarged on the right panel is the overlay between the predicted (green) and the experimentally solved (gray, PDB ID: 6IIE [32]) structures of the EF hand domains. The experimentally solved structure had Ca^2+^ bound, which is shown in purple. The red arrow indicates the putative ATP-binding site is exposed in the predicted structure.

A pocket between the catalytic and accessory domains is believed to be the putative active site of the enzyme. This region in DGKα exhibits a high degree of conserved residues when comparing the sequence of the ten human DGK paralogues (Figure 2A). This putative active site has a positively charged electrostatic surface and ATP, which is negatively charged, docks into this pocket (Figure 2B). Indeed, recently it has been shown by in silico studies that this region is a conserved ATP-binding pocket in all ten human DGK paralogues [30]. Of the three type I DGKs, DGKα is the only one that is sensitive to changes in physiological levels of Ca^2+^, the other two paralogues have a higher affinity for Ca^2+^ and always have Ca^2+^ bound in vivo. It has been shown that the binding of Ca^2+^ to the EF hand motifs in DGKα triggers a conformational change in the enzyme N-terminus, which exposes active and hydrophobic sites promoting membrane binding [31]. The tridimensional high-resolution structure of the isolated EF hand motifs of DGKα bound to Ca^2+^ has been solved experimentally by X-ray crystallography [32]. When the structure of the isolated domains is overlapped with the predicted structure of the full-length enzyme, there is high degree of structural homology (Figure 3). In addition, the EF hand motifs are located away from the putative active site suggesting that the predicted structure could be the active form of the enzyme.

### 2.3. Subcellular Localization

DAG and PA are both lipid molecules, which have low water solubility and are mainly found embedded into the membrane. Hence, membrane interaction is a requirement for DGKα enzymatic catalysis. In resting cells, DGKα is found mainly in the cytosol [36]. However, upon cell stimulus DGKα has been described to show a rapid and transient partition to different biological membranes. For instance, activation of T-cell antigen receptor (TCR) or tyrosine kinase receptors triggers binding of DGKα to the plasma membrane (PM), which is dependent on both calcium binding to the EF hand domain of DGKα and Src (proto-oncogene tyrosine protein kinase)/Lck (lymphocyte-specific protein tyrosine kinase)-dependent phosphorylation of Tyr^335^ [12,17,28,37,38,39,40,41,42,43]. In lymphocytes, recruitment of DGKα to PM has also been shown to occur in response to an increase in the levels of phosphatidylinositol-3-kinase (PI3K) lipid products, i.e., phosphatidylinositol-3,4,5-triphosphate (PI-3,4,5-P_3_) and phosphatidylinositol-3,4-bisphosphate (PI-3,4-P_2_) [42]. In addition, upon TCR activation, DGKα has also been found associated with intracellular membranes involved in the secretory vesicle pathway, e.g., trans-Golgi network (TGN), endosomes, multivesicular bodies (MVB), and exosomes [44,45,46]. Furthermore, interleukin 2 (IL2) stimulation of T cells, which promotes proliferation of T cells, triggers DGKα translocation to the perinuclear region [36]. It, thus, seems that DGKα is recruited to different membranes in a stimulus-specific context. Therefore, DGKα could access different pools of DAG, which, in turn, would lead to its role in different biological processes.

### 2.4. Modulation of DGKα by Membrane Physicochemical Properties

**Figure 4:**
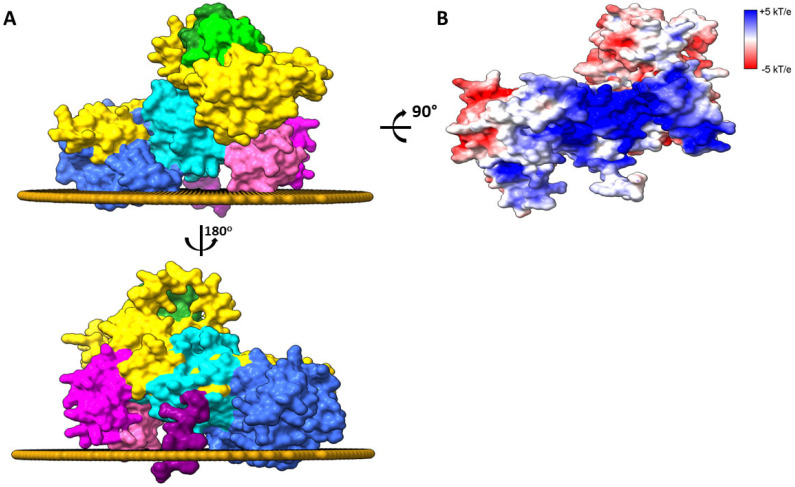
Membrane-bound DGKα. (**A**) The spatial arrangement of the AlphaFold predicted structure of human DGKα bound to the eukaryotic plasma membrane was done with PPM 3.0 [47]. Residues in C1 and accessory domains as well as the Pro segment anchors the enzyme to the membrane interface. Only the membrane leaflet interacting with the enzyme is shown. Structures were generated using Chimera X [26] and domains colored as follows: EF hand 1 (residues 114–142, lime), EF hand 2 (residues 159–187, forest green), C1A (residues 206–253, hot pink), C1B (residues 268–319, magenta), catalytic (residues 376–500, dark turquoise), accessory (residues 520–701, royal blue), and Pro (residues 722–735, purple). The remaining protein was colored in gold. (**B**) The surface of the enzyme interacting with the membrane is shown and colored by Electrostatic surface potential (ESP, red = acidic, white = neutral, and blue = basic surfaces). The membrane leaflet interacting with the enzyme was removed for clarity.

The description at the molecular level on how DGKα interacts with the membrane is not fully understood yet. However, it has been shown that membrane binding in intact cells is dependent on the C1 domains and the Pro-rich segment on the enzyme C-terminus [40]. Indeed, positioning of the Alphafold predicted tridimensional structure of DGKα on a model of mammalian PM shows that the energetically optimal spatial position involves interaction of DGKα C1 domains and Pro-rich segment at the C-terminus as well as the accessory domain with the PM (Figure 4A). The predicted interaction between DGKα and the membrane is energetically favored (ΔG^water→membrane^ ≈ −13 kcal/mol) and the enzyme has a shallow insertion (~7 Å) into the membrane hydrophobic core. In this spatial arrangement, the surfaces of the catalytic and C1 domains laying juxtaposed to the membrane is positively charged (Figure 4B). This would favor an electrostatic interaction with negatively charged lipids, which might approximate the catalytic site to the membrane interface, which, in turn, would promote enzymatic catalysis. This agrees with the fact that negatively charged lipids activate the enzyme (see below).

DGKα enzymatic properties are highly sensitive to the physicochemical properties of the membrane the enzyme binds to. It has been shown that major lipid components of the PM, one of the main biological membranes where DGKα exerts its biological function (see above), could either inhibit or activate DGKα enzymatic activity. For instance, sphingomyelin has been shown to inhibit DGKα in the presence of Ca^2+^ [48]. In the absence of Ca^2+^, DGKα can be activated by phosphatidylethanolamine (PE) and cholesterol [48]. In addition to these zwitterionic/neutral lipids, an anionic lipid enriched in the PM, i.e., phosphatidylserine (PS), has also been shown to activate DGKα [28,49]. It has been proposed that PE and cholesterol might induce a conformational change on the enzyme N-terminus, which, in turn, would expose the enzyme active site in a similar fashion to the one induced by Ca^2+^-binding to EF hand motifs [48]. On the other hand, PS activation has been proposed to be due to an interaction with the catalytic domain, which might serve to orient the catalytic site [49]. In addition to major lipids, potent lipid second messengers that are often present in low amounts at the membrane have been reported to regulate DGKα. It has been shown that PI-3,4-P_2_, PI-3,4,5-P_3_, and sphingosine activate DGKα [42,50,51,52]. Interestingly, while these PIPn are highly negatively charged, sphingosine is a positively charged lipid. Therefore, it seems that the modulations of DGKα by these lipid second messengers are occurring by interaction with different regions of the enzyme.

The activity of DGKα is sensitive to the lipid composition of the membrane the enzyme binds to. However, lipid composition does not seem to affect the DAG acyl chain specificity of the enzyme [53,54]. On the other hand, DGKα DAG acyl chain specificity seems to be modulated by membrane physical properties, i.e., to the shape of the membrane the enzyme binds to [23]. It has been shown that DGKα exhibits no preference for the molecular species of DAG when assayed in detergent micelles or in flat planar bilayer membranes. For instance, when the enzymatic activity was assayed in liposomes using four different DAG molecular species of DAG (i.e., 18:0/20:4-DAG, 18:1/18:1-DAG, 16:0/18:1-DAG, and 16:0/16:0-DAG), DKGα exhibited similar affinity as well as catalytic turn-over for all four DAG molecular species, indicating the enzyme bears no DAG acyl chain specificity in a flat planar bilayer. However, when the membrane phase is changed to increase the physical curvature of the interface, there is a preference for DAG species with saturated/monounsaturated acyl chains [23]. For instance, the enzymatic activity in curved membranes was found to be ca. 10-fold higher in comparison to flat membranes if the DAG species were either 16:0/18:1-DAG or 16:0/16:0-DAG, while the activity only increased ca. 2-fold if the DAG species were either 18:0/20:4-DAG or 18:1/18:1-DAG. These are the same molecular species found to be specific for DGKα activity in cells [19,21,22]. Thus, the substrate specificity depends on the shape of the membrane to which the DGKα is bound. This phenomenon is independent of membrane curvature bending strain but is dependent on membrane shape and possibly on the presence of Gaussian curvature [23]. An analogous effect of membrane shape on substrate preference was initially shown for DGKε [55]. Although it has been appreciated for many years that some biological processes are sensitive to curvature at a large length scale where curvature strain would not be present, it had not been demonstrated at the level of molecular events [56]. This has changed since the reports that DGKα/ε substrate acyl chain specificity is modulated by membrane shape and not curvature strain [23,55]. Hence, membrane shape modulation of enzymatic properties is a modern concept, which is proposed to be broadly found in different biological processes.

## 3. DAG and PA: Intermediates of Lipid Metabolism, Membrane Curvature Generators and Signaling Molecules

DAG and PA are lipids present in low levels in biological membranes. However, upon cell stimulus, their levels are rapidly and transiently increased [57,58,59]. The levels of these lipids are highly regulated since both lipids are: (i) key intermediates in lipid metabolism, (ii) precursors for the biosynthesis of new lipids, (iii) important for the generation of membrane curvature, and (iv) potent signaling molecules. Hence, both lipids are critical for maintaining cellular homeostasis [58,59,60].

DAG and PA are key intermediates in the metabolism of glycerophospholipids (e.g., PA, PE, phosphatidylcholine—PC, phosphatidylinositol—PI, PS) and triacylglycerols [58]. DAG and PA also play a role in modulating the physicochemical properties of the membrane they are embedded into. DAG has a hydroxyl group as the headgroup and, therefore, it is a neutral lipid (Figure 1A). On the other hand, PA has a phosphate group as a headgroup and, consequently, it is negatively charged (Figure 1A). PA has a second pKa around physiological pH and, therefore, it can exist as an equilibrium between mono- or divalent anionic species [57]. Each of these lipids are present in cells as a variety of molecular species due to differences in length (i.e., 14–22 carbon atoms long) and unsaturation (i.e., 0–6 unsaturation) of the acyl chains. These differences in the chemistry of these lipids contribute to the different interactions with downstream signaling molecules. In addition, both lipids present a conical molecular shape, which makes them inducers/stabilizers of membrane negative curvature [61,62]. Their molecular shape enables the penetration of proteins into the membrane–water interface by decreasing the lipid packing at the headgroup region. Indeed, they are often found to regulate biological processes where large membrane morphological changes take place, where they facilitate vesicle budding and fusion [63].

DAG signaling is important for a variety of cellular processes including cell proliferation, survival, motility, and membrane trafficking and secretion [64]. DAG triggers downstream signaling by recruiting proteins containing DAG binding motifs, i.e., C1 domains, to the membrane. Proteins that are regulated by DAG include protein kinase C (PKC), protein kinase D (PKD), Ras guanyl nucleotide-releasing protein 1 (RasGRP1), and chimaerins [64]. Recently, it has been shown that PKC is sensitive to the nature of the DAG molecular species, i.e., different PKC bind different DAG molecular species in live cells [65]. Contrary to the well-established role of DAG in cellular signaling, PA signaling was long believed to occur, but the lack of the identification of PA-binding domains in proteins led to an elusive role. More recently this has changed, and it has been shown that PA can regulate a variety of biological processes due to the regulation of proteins involved in cell growth, differentiation, migration, and membrane trafficking [20,59]. Proteins regulated by PA include mammalian target of rapamycin (mTOR), PKCε/ζ, phospholipase C (PLC) β1/γ1, Ras GTPase activating protein (RasGAP), chimaerin, ADP-ribosylation factor 1 (Arf1), and Ras-related C3 botulinum toxin substrate 1 (Rac1) [20,59]. It also seems that some of these proteins present specificity for the molecular species of PA they bind to [20]. For a long time, DAG/PA cellular signaling was believed to be dependent on the regulation of their levels in different biological membranes. This paradigm seems to be challenged by the identification of proteins that have specificity for different molecular species DAG/PA. It thus seems reasonable to propose that contrary to the long-believed concept, it is currently thought that DAG/PA signaling is not solely a result of change in their concentrations, but also of the change in the concentration of specific molecular species of DAG/PA.

## 4. DGKα in Cell Biology

### 4.1. T Cells

Both DAG and PA are potent lipid signaling molecules. Therefore, most of what is known about the role of DGK in cell biology is a consequence of either attenuation of DAG and/or intensification of PA signaling pathways. Since DGKα is highly expressed in T cells, its roles in T cell biological processes have been best characterized [66]. For instance, DGKα controls T cell polarity at the immunological synapse (IS), i.e., at the interface between antigen-presenting cell (APC) and T cells [67]. Upon TCR activation, a signaling platform is formed in response to membrane remodeling and organelle assembly (e.g., Golgi apparatus, secretory lysosomes, mitochondria) at the inner leaflet of the PM of T cells around the IS [67]. Polarization of the microtubule organizing center (MTOC) of T cells towards the IS allows directionality in the secretory pathway towards the APC. In this process, it has been shown that DGKα controls MTOC polarity by limiting the diffusion of DAG (through its conversion into PA) from the periphery of the IS, where it is preferentially localized likely via a specific interaction with PIP_3_ [68]. DGKα has also been shown to play a role in T cell proliferation, whereupon T cell stimulation with IL2 DGKα translocates from the cytosol to the perinuclear region, where the production of PA by DGKα has been associated with its mitogenic properties [36]. In addition to these positive effects of DGKα in T cell biology, DGKα has also been shown to exhibit negative effects on T cells, often through its increased expression and, consequently, decrease in DAG signaling. For instance, in the absence of costimulatory signals or weak activation of TCR, DGKα expression is increased and, consequently, there is an attenuation of DAG signaling at the PM [15,17]. This leads to a hyporesponsive state in T cells, namely anergy [69]. This is a consequence of decreased recruitment of RasGRP1 to the PM, which is dependent on DAG, and, therefore, inactivation of extracellular signal-regulated kinase (ERK) signaling pathway [16,18]. In addition, DGKα overexpression downregulates secretory vesicular trafficking at the IS in T cells. Upon TCR activation, T cells can secrete exosomes through a series of membrane fusion/fission events, which starts with vesicle budding at the TGN and ends with the fusion of MVB with the PM to secrete exosomes containing soluble factors towards the target cell. It has been shown that increased DGKα expression downregulates the formation of MVB and exosomes secretion [44,45,46]. It has been proposed that this is a consequence of DGKα kinase activity at the TGN, which, in turn, inhibits PKD, a key enzyme to control secretory vesicle budding in the TGN, and, therefore, the formation of MVB [44,45,46].

It is important to mention that DGKα is not the only paralogue that is highly expressed in T cells, DGKζ is also highly expressed and, therefore, DGKζ also plays a role in the biology of T cells by regulating DAG/PA levels. DGKα and DGKζ show redundant and specialized roles in T cell biology. For instance, both DGKα and DGKζ have been shown to modulate DAG levels at the IS, which is critical for proper organization of the signaling platform [68,70,71]. On the other hand, expansion of innate-like cytotoxic T lymphocytes mediated by cytokine and independent of antigen stimulation is predominantly limited by DGKζ activity [72]. The study of the molecular mechanisms governing the function of DGKα and DGKζ in T cell biology is a field of increased interest. However, in this review, the primary focus is on DGKα and the reader is referred to excellent reviews addressing the topic [66,73,74]. Nevertheless, it will be of interest to evaluate the modulation of DGKζ enzymatic activity and/or DAG substrate acyl chain specificity by membrane shape and how this compares to DGKα. This might help to clarify the redundant/specialized roles of these enzymes in T cell biology.

### 4.2. Cancer Cells

DGKα is also highly expressed in various cancer cells, where it has been shown to be involved in cancer cell survival, proliferation, migration, and invasiveness [9,10,11,12,75]. While DAG attenuation has been associated with the majority of DGKα roles in T cell biology, it seems that the role of DGKα in cancer cell biology is mainly associated with PA production. In human hepatocarcinoma, it has been shown that DGKα is associated with tumor progression by activation of the mitogen-activated protein kinase (MAPK) pathway [13]. In melanoma, DGKα has been shown to inhibit apoptosis by activating tumor necrosis factor α (TNFα)-induced activation of nuclear factor kappa-light chain enhancer of activated B cells (NF-κB), which was proposed to be a result of DGKα-produced PA activating PKCζ-mediated phosphorylation of S^311^ of NF-κB p65 [10,76]. In glioblastoma cells, DGKα production of PA has been associated with inhibition of apoptosis and regulation of hypoxia-inducible factor 1-α (HIF1-α) and mammalian target of rapamycin (mTOR) oncogenic pathways [14,75]. In ovarian cancer, it has been shown that DGKα promotes platinum resistance by producing PA, which activates the transcription factor c-JUN and, consequently, enhancing the transcription of cell-cycle checkpoint regulator WEE1 gene [77]. In esophageal squamous cell carcinoma, PA production by DGKα has been associated with the activation of Akt/NF-κB signaling, which, in turn, reduced cAMP levels and PTEN activity leading to tumor progression [78]. DGKα has also been shown to promote tumor invasion and progression by generating PA at invasive pseudopods triggering the localization of atypical PKC, which control Rac-mediated protrusion elongation and Rab-coupling protein (RCP)-dependent integrin recycling [79,80]. In several cases, inhibition of DGKα in the cancer models described above has been associated with antitumor properties.

## 5. Membrane Morphology Changes during Cancer and T Cell Biological Processes: Implications for DGKα Enzymatic Properties

Signaling events are highly regulated spatial-temporal events that require signal specificity (to minimize crosstalk), efficiency (to avoid waste of cellular energy), and directionality (to ensure the information is directed from stimulus to response). As it is described above, the role of DGKα in T cell and cancer biology often occurs by the modulation of DAG/PA signaling. As such, one would expect that DGKα is directed to specific membrane regions upon cell stimulus, where it can exert its biological function in a specific, efficient, and directional manner. It is interesting to note that in several biological processes where DGKα has been reported to play a role membrane remodeling is often taking place. It seems that in these processes, DGKα is associated with membrane regions bearing high curvature. Membrane regions with high curvature endow DGKα with a DAG acyl chain specificity [23]. Therefore, it seems reasonable to propose that whenever DGKα is involved in membrane remodeling, it is not only modulating the levels of DAG/PA, but rather specific molecular species of these lipids, which, in turn, would add an extra layer of specificity in those signal events. DGKα, then, could act as a bridge between large membrane morphological changes and the regulation of specific molecular lipid species of DAG/PA and, consequently, trigger specific signaling pathways. In the following paragraphs, a discussion of a few examples of the interplay between DGKα and membrane remodeling events in T cell and cancer biology will be discussed.

### 5.1. TCR Activation

**Figure 5:**
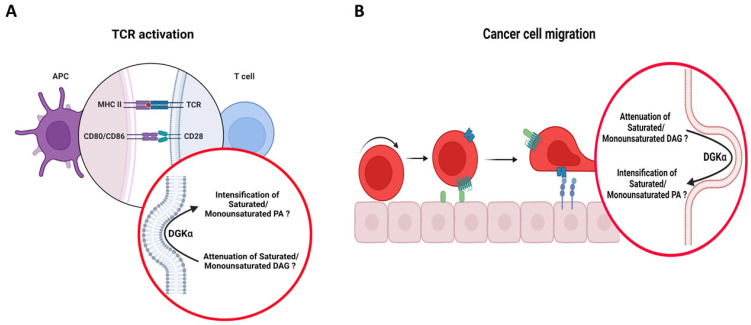
Putative role of DGKα in bridging membrane morphological changes to specific molecular species of DAG/PA in T cell and cancer biological processes. Cartoons illustrating (**A**) TCR activation and (**B**) cancer cell migration. It is proposed that in these processes, DGKα would localize in membranes bearing negative curvature (around the IS during TCR activation and the tips of invasive protrusions during cancer cell migration), which would endow the enzyme with its acyl chain specificity, letting it act on its preferred substrates, i.e., DAG with saturated/monounsaturated acyl chains. Schematic representations were generated using Biorender (©BioRender-biorender.com).

Upon TCR activation, there is formation of a T cell-APC contact zone with specialized structure, namely IS [81]. The formation of the IS is an important step in the immune response, which guarantees T cell-APC communication and ongoing T cell signaling and activation. The union between these two cell types involves major rearrangements of signaling molecules and cell shape [82]. The IS orchestrates downstream signaling events by driving the accumulation and partition of synaptic components in segregated discrete supramolecular activation clusters (SMACs), which are radially symmetric compartments [83,84,85,86]. At the central SMAC (c-SMAC) there are accumulation of TCR and costimulatory molecules (e.g., CD28), on the periphery of SMAC (p-SMAC) is a region characterized by adhesion molecules (e.g., LFA-1—lymphocyte function-associated antigen-1, integrin αLβ2), which is followed by a distal zone of SMAC (d-SMAC) enriched in actin filaments [87]. In this process, DGKα has been reported to localize at the p-SMAC where it was shown to play a role in MTOC polarity by limiting DAG signaling to the periphery of the IS [88]. A tension on the membrane caused by differences in the dimensions of complexes at the T cell-APC cell–cell contact zone leads to membrane bending at the interface between p-SMAC and c-SMAC [89]. On the other side of p-SMAC, at d-SMAC, actin polymerization also pushes the membrane to bend it [87]. The change in membrane morphology around p-SMAC yield membrane regions with high negative curvature at the cytoplasmic surface of the PM in T cell. This type of membrane morphology change is the one that has been shown to endow DGKα with its DAG acyl chain specificity [23]. Hence, it is tempting to speculate that in this process, DGKα is not only regulating the gradient of DAG at p-SMAC, but rather regulating the gradient of a specific pool of DAG (i.e., DAG enriched with saturated/unsaturated acyl chains) (Figure 5A). This is consistent with the report that different molecular species of DAG can activate different forms of PKC and, therefore, trigger different signaling pathways in live cells [65]. While this is a reasonable assumption, it is currently unknown whether the DAG metabolized by DGKα at the IS has a specific molecular species profile. Future experiments might pave the way to better understanding of the role of DGKα in the polarization of MTOC at the IS.

At the IS, vesicular trafficking plays an important role in the IS assembly and function [90,91]. For instance, during TCR activation, continuous endocytosis of engaged as well as bystander TCR removes those from the c-SMAC region of the plasma membrane [92,93,94]. Replenishment of TCR at the c-SMAC occurs via polarized endosomal recycling towards the IS, which is critical for sustained signaling at the IS during the timeframe required for T cell activation [95]. Intracellular trafficking also plays a key role in the polarized secretion of different cargoes towards the APC [96,97,98,99,100]. During vesicular trafficking, large membrane morphological changes take place, which are determined by membrane fusion/fission events during endocytosis/exocytosis. This type of membrane remodeling could modulate DGKα. Indeed, it has been shown that DGKα plays a negative role in the secretion of exosomes upon T cell stimulation. For instance, upon T cell activation and expansion, they can be eliminated via the activation-induced cell death (AICD) program, which plays a role in T cells homeostasis and immune tolerance [101]. AICD is dependent on upregulation of proapoptotic Fas ligand (FasL) gene and its secretion into exosomes [102]. Exosomes are small, single membrane vesicles secreted by a variety of cells, which originate from the endosomal system, more specifically by the inward budding of the limiting membrane of late endosomes during the maturation process [103]. The accumulation of intraluminal vesicles inside endosomes leads to the formation of MVB, which upon cell stimulation fuse with PM leading to the secretion of exosomes. Overexpression of DGKα has been reported to inhibit the formation of MVB and, consequently, secretion of exosomes upon T cell stimulation [44,45,46]. In this process, it has been proposed that DGKα acts by controlling the inward budding in MVB, where the membrane acquires high negative curvature. This suggests that DGKα could be doing so by controlling specific molecular species of DAG. In addition, it has been shown that in tumor infiltrating CD8 cells, increased DGKα activity led to defects in the exocytosis of granules and lytic function of these cells [104]. Hence, it seems that DGKα is involved in the vesicular trafficking at the IS by negatively impacting granule docking/fusion and/or release. In neurological synapse, DAG is involved in synaptic vesicle priming and docking [105]. It is tempting to speculate that the negative role of DGKα during exocytosis at the IS might be related with the metabolism of DAG, which, in turn, would impact on vesicle priming/docking and/or release. It would be interesting to evaluate the substrate acyl chain specificity of DGKα as well as the role of specific molecular species of DAG/PA in the regulation of this process in the future.

### 5.2. T Cell Anergy

In the absence of a costimulatory signal or weak TCR activation, DGKα has also been shown to lead to T cell anergy [15,17,69]. It has been proposed that this is a consequence of increased DGKα activity decreasing the level of PM DAG and, consequently, the downstream signaling pathways needed for T cell activation. A lack of proper membrane bending could also play a role in this process. For instance, in the absence of costimulatory signal or weak TCR activation one would expect that the tension at PM of T cells caused by complexes at the T cell–APC cell–cell contact zone and/or the cytoskeleton in the T cell would be lower. This, in turn, would decrease membrane bending and the generation of high membrane curvature around p-SMAC. In flat membranes (in relation to the size of DGKα), it has been shown that DGKα lacks substrate acyl chain specificity [23]. Hence, it is reasonable that in the absence of costimulatory signal or weak TCR activation, the required membrane morphological changes needed to endow DGKα substrate acyl chain specificity do not occur. This would, then, lead to DGKα acting more broadly on PM DAG and, consequently, contributing to a decrease in DAG levels rather than on specific molecular species of DAG. The decreased DAG levels would inhibit downstream signaling pathways needed for T cell activation and, therefore, lead to T cell anergy. Future experiments focusing on the relationship between membrane remodeling and the substrate acyl specificity of DGKα will help broaden the understand of the molecular mechanisms governing the role of DGKα in the T cell anergy.

### 5.3. Cancer Cell Migration 

The invasion and metastasis of cancer cells are processes that rely on cell migration [106]. A change in the shape and stiffness of the cell is a requirement for cell migration. Cell migration is a consequence of five interdependent cyclic steps: (i) formation of cell protrusions/extensions on the leading edge, (ii) formation of focal contacts between cell and extracellular matrix (ECM), (iii) focalized proteolysis on the ECM, (iv) cell contraction by actomyosin, and (v) detachment of the trailing edge [106,107]. The formation of cell protrusions/extensions (e.g., lamellipodia, filopodia, pseudopod, ruffles, and spikes) results from actin polymerization and the recruitment of a variety of structural and signaling molecules at the cell leading edge, which yield a morphological change on the PM and dynamic interactions with the ECM [106]. In this process, it has been shown that DGKα plays a role in the formation and elongation of invasive protrusions. Upon growth factor or chemokine stimulation, DGKα binds to the PM leading to the production of PA at the tips of invasive pseudopods [39,79,80,108,109,110]. The DGKα-produced PA, then, acts to dock atypical PKCζ/ι, Rab11 family of interacting protein 1 (Rab11-FIP1), and integrin α5β1, which triggers actin polymerization, elongation of invasive protrusions, and polarize vesicular trafficking as well as promote directional migration [80,108,109]. In invasive pseudopods, actin polymerization at the cell leading edge leads to an outward directed force that creates membrane tension and, therefore, cause membrane bending [111]. Therefore, the cytoplasmic face of the tip of invasive pseudopods presents membrane negative curvature. Since this type of membrane curvature provides a suitable platform for DGKα substrate acyl chain specificity, one would expect that the DAG/PA molecular species profile in this process would be favoring the saturated/monounsaturated acyl chains (Figure 5B). However, at least in human ovarian cancer cell line, it seems that DGKα exhibits specificity for 38:4 (likely, 18:0/20:4) DAG species during invasive migration [79]. There are two possible interdependent explanations for this. First, PLCγ is believed to contribute to DAG enrichment at the leading edge via the metabolism of PIP_2_ upon growth factor stimulation [112]. PIP_2_ in mammals is enriched in 18:0/20:4 acyl chains [113]. Hence, it might be that the observed preference shown for DGKα in this process is a consequence of preferential enrichment on 18:0/20:4 DAG at the invasive protrusions. In addition, it is also possible that DGKα is acting on DAG in a region of the membrane where curvature is not large enough to endow its substrate acyl chain specificity and then, the PA is diffusing towards the tip of the invasive protrusions. However, caution is warrant in the analysis of this lipidomic studies as (i) they were done in whole cells and, therefore it is difficult to pinpoint the specificity at the tip of the invasive protrusions; and (ii) another paralog, i.e., DGKι, might have also contributed to those results [79]. Hence, the role of DGKα in bridging membrane morphology with the regulation of specific molecular species of DAG/PA in invasive protrusion during tumor migration requires further studies.

### 5.4. Nuclear Envelope

DGKα has been observed to translocate from the cytosol to the nucleus upon both non-proliferative (serum starvation) and proliferative stimulus, where it has been proposed to regulate cell cycle [21,36,114]. However, the molecular mechanism and possible signaling role of DGKα at the nuclear envelop is not yet well established. At the fusion between inner and outer nuclear membranes, there are nuclear pore complexes (NPC), which generate nuclear pores. Nuclear pores are the only route for polar substances to translocate across the nuclear membrane. Nuclear pores are among the largest structures found in eukaryotic cells. Each nuclear pore is composed of 500 to 1000 copies of nucleoporin protein as well as PA that accumulate at stalled nuclear pore complexes [115]. Recently, the architecture of the nuclear pore has been described at high resolution [116,117]. In T cells and cancer cells, serum starvation led to the specific production of saturated and monounsaturated 16 carbon atoms PA species by DGKα, suggesting that these molecular species of PA might have been produced in the nucleus in non-proliferative conditions [19,20,21]. Interestingly, these are the molecular species of DAG that DGKα shows substrate acyl chain specificity upon membrane morphological changes [23]. Hence, it is seeming tempting to propose that in non-proliferative conditions, DGKα might be acting on nuclear membranes presenting high negative curvature, such as those found at the nuclear pore region. While PA production by DGKα in the nucleus of T cells has been associated with IL2-mediated lymphocyte proliferation, whether DGKα exhibits any substrate acyl chain specificity in this process is not known yet.

## 6. Specificity of DGKα Effectors Dependent on DAG/PA Molecular Species

The finding that DGKα exhibits substrate acyl chain specificity raises the question of whether the function of DGKα effectors depend on the molecular species of DAG/PA. While this is a topic that starts to emerge and needs future studies to focus on this aspect, there is some evidence that some DGKα effectors depend on the molecular species of DAG/PA. For instance, the main role of DGKα in T cells has been ascribed to the regulation of DAG metabolism downstream of TCR activation via the modulation of RasGRP1, a guanine nucleotide exchange factor that activates Ras/ERK signaling [17,18,37]. RasGRP1 is a cytosolic protein that binds to membranes upon DAG generation. RasGRP1 has been described to partition to the Golgi where is inactive and to the PM where it is active [118,119,120,121]. Although the effects of only two DAG molecular species have been evaluated, it has been shown that RasGRP1 bears specificity for DAG molecular species of DAG in vitro, binding more strongly to DAG bearing monounsaturated acyl chains (i.e., 18:1/18:1-DAG) in comparison to polyunsaturated DAG (i.e., 18:0/20:4-DAG) [118]. While none of these DAG molecular species are those reported to be the ones DGKα has specificity to, the specificity in the DAG binding suggests RasGRP1 has specificity for DAG molecular species and urge for a more systematic study in the future. In hepatocellular carcinoma, it was shown that DGKα is involved in the tumor progression by activation of the MAPK pathway via modulation of Raf-1 (C-Raf) [13]. Raf-1 is known to interact with PA, showing specificity for PA molecular species containing monounsaturated acyl chains (i.e., 16:1/18:1-PA and 18:1/18:1-PA) in comparison to saturated acyl chains (i.e., 16:0/16:0-PA) [122,123,124]. In glioblastoma, DGKα has been associated with tumor proliferation via regulation of mTOR expression and activity [14]. DGK-derived PA is known to modulate the activity of mTOR [125,126]. mTOR is found in two structurally and functionally distinct multiprotein complexes, mTOR1 and mTOR2 [127]. It has been shown that mTOR2, which mediates spatial control of cell growth by regulating the actin cytoskeleton, binds more strongly to PA containing saturated acyl chains (i.e., 16:0/16:0-PA) than saturated/monounsaturated PA (i.e., 16:0/18:1-PA and 18:1/18:1-PA) [128]. In cells, DGKα has been shown to exhibit DAG acyl chain specificity towards 16:0 and 16:1 acyl chains and, therefore, leads to the suggestion that DGKα might modulate Raf-1 and mTOR2 by producing these PA molecular species [19,20,21,22]. Overall, it seems that some DGKα effectors are dependent on DAG/PA molecular species. It will be interesting in the future to study the specificity of these effectors for different DAG/PA molecular species more systematically as well as in which cellular context this specificity is relevant for signaling. This might lead to a better understanding of the modulation of these effectors by DGKα, which, in turn, could broaden our understand of the role of DGKα in cancer and immunosurveillance.

## 7. Therapeutic Approaches Targeting DGKα in Cancer and Immunosurveillance

**Figure 6:**
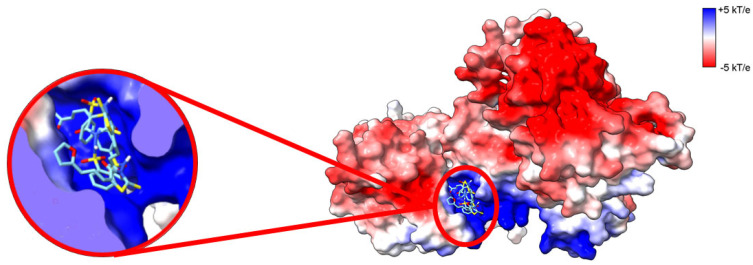
CU-3 inhibits DGKα by binding into the ATP-binding site. Electrostatic surface potential (ESP) of AlphaFold predicted high-resolution structure of human DGKα. ESP was calculated with adaptive Poisson–Boltzman solver (APBS) [34] and colored according to color key (red = acidic, white = neutral, and blue = basic surfaces). Enlarged on the left panel CU-3 docked to DGKα. CU-3 docks into the putative ATP binding site with an average affinity of −8.0 ± 0.3 kcal.mol^−1^. The three lowest energy docking poses are shown. Blind docking was performed with Webina [35].

The association between DGKα and cancer as well as its role in immunosuppression (i.e., T cell anergy) has attracted great attention to DGKα as a potential molecular target in immunotherapy. Since the role of DGKα in these biological processes has been associated with increased expression of DGKα, studies have focused on the effect of downregulating DGKα expression or inhibition of its kinase activity. For instance, inhibition of DGKα expression via molecular genetics techniques such as the use of small-interfering RNA against DGKα or the use microRNA-297 showed promise as evidenced by the suppression of the proliferation of colon and breast cancer as well as glioblastoma [11,75]. The toxic effect is selective to cancer cells as only cancer cells (not normal cells) showed increased apoptosis by downregulation of DGKα activity [14]. In addition to molecular genetic techniques, the use of inhibitors against DGKα activity has been investigated. One of the challenges in developing inhibitors of DGK, it is the specificity due to the multiplicity of DGK in humans. Nevertheless, the use of allosteric inhibitors with some specificity for DGKα, such as R59022, R59949, and ritanserin, has been reported [129,130]. These inhibitors have a half inhibitory concentration (IC_50_) of ca. 20 μM [129,130]. One of the disadvantages of these inhibitors is that they also inhibit other DGK paralogs. For instance, R59949 inhibits DGKγ with similar IC_50_ to DGKα and a slightly higher IC_50_ to DGKθ and DGKκ [129,130]. Recently, a mass throughput screening has led to the identification of a putative competitive ATP inhibitor with improved selectivity for DGKα, namely CU-3 [21,131]. CU-3 has been shown to have a low IC_50_ for DGKα, i.e., 0.6 μM, while its IC_50_ for other DGK was > 5 μM [131,132]. Blind docking of CU-3 to DGKα leads to the compound being docked to the putative ATP binding site in the AlphaFold predicted structure of DGKα (Figure 6). This is interesting as it has been recently reported that the putative ATP binding site is structurally conserved among human DGK [30]. This suggests that although the ATP binding site is structurally similar among human DGK, there might be some differences in the residues lining the surface of the ATP binding site of DGKα in comparison to other paralogs that yields the specificity of CU-3. CU-3 has been shown to induce apoptosis in several cancer cells as well as enhancing immune response [21,131]. In addition, CU-3 combined with immune checkpoint inhibitors were observed to have the dual effect of both inhibiting the growth of hepatocellular carcinoma as well as increasing the immunological surveillance by T-lymphocytes [132]. Hence, suppression of DGKα activity has the promise of having a dual activity against cancer by both unleashing T cell activity and inhibiting proliferation of cancer cells, the latter being proposed to be a consequence of increased apoptosis of these cells.

## 8. Conclusions

DGK is known to play critical roles in cellular signal transduction. This is because DGK enzymatic activity switches the signaling from DAG to PA. For a long time, this was believed to be a consequence of changes in the levels of these lipids. However, the identification of substrate acyl chain specificity for different DGK paralogs has challenged this paradigm, suggesting that DGK can regulate DAG/PA signaling not only by changing the level of these lipids, but also by acting on specific molecular species of DAG/PA. In the case of DGKα (and DGKε), it has been shown by in vitro studies that large membrane morphological changes can act as a factor in controlling the substrate acyl chain specificity of the enzyme. Interestingly, as outlined above, in several biological processes where DGKα has been reported to play a role, the membrane undergoes large morphological changes. Hence, it is reasonable to propose that in these biological processes, DGKα not only regulates DAG/PA levels, but also exhibits specificity in the regulation of particular molecular species of DAG/PA. Although this is reasonable based on the well demonstrated in vitro studies, it is currently not known if membrane morphological changes in a biological membrane will result in a change in the substrate specificity of DGKα. Here, one of the challenges is characterizing the DAG/PA molecular species profile in the different membranes which DGKα has been reported to be acting on. Lipidomic studies are done in whole cells. This is based on the challenges in the purification of biological membranes. However, whole cell lipidomic will not help answer this question. To address this, one will need use high resolution spatial-temporal techniques. For instance, mass microscopy would allow the acquisition of high-resolution imaging mass spectrometry and, therefore, the characterization of DAG/PA molecular species profile in different membranes without the need of membrane purification. In addition, the use of super resolution microscopy could provide spatial-temporal information of this process, which could be done by the development of probes specific for different molecular species of DAG/PA. One caveat though is the presence of multiple paralogs of DGK in most cell types as well as the presence of other DAG/PA-metabolizing enzymes that will affect the DAG/PA molecular species profile. In this case, control experiments with different DGKα constructs (kinase dead, constitutively active), silencing/inhibition of DGKα activity, as well as DGKα labeling for super resolution microscopy are needed to help corroborate the findings. Future studies addressing these challenges might help build a more global understanding of the molecular mechanisms governing the biological role of DGKα, specifically in cancer and immunosurveillance.

In the last decade, DGKα has gathered great attention as a potential target in immune therapy. This is because the silencing of its activity provides a two-pronged attack on cancer cells. The inhibition of DGKα prevents cancer cell proliferation by promoting apoptosis of cancer cells. Independently of this, inhibition of DGKα also releases T-cells from anergy and allows for greater immune clearance of cancer cells. One of the main challenges is the design of inhibitors with high affinity (low IC_50_) and specificity (due to the multiplicity of DGK in humans). One aspect to consider regarding specificity is the fact that some mammalian DGK presents substrate acyl chain specificity. Most of the inhibitors currently available seem to target ATP-binding. All DGK binds ATP as this is a requirement for their catalytic activity. It has been shown that the ATP binding pocket in human DGK are structurally conserved. Hence, an alternative approach might be to focus on inhibitors that target DGK substrate acyl chain specificity. In the case of type I DGK, it has been shown that the C1 domains are the structural motifs on these enzymes responsible for DAG substrate acyl chain specificity. In the future, the design of DGKα inhibitors might want to target the substrate acyl chain specificity at the C1 domains. While work is on the way to experimentally determine the high-resolution structure of DGKα, validation of the high-resolution predicted structure of DGKα might help pave the way for the development of better, more potent, and specific inhibitors of DGKα with the aim to be used in immunotherapy. This will have the potential to improve the efficacy and broaden the use of immune therapy, which, in turn, will improve the health outcomes of cancer patients.

## Abbreviations

AICD	activation-induced cell death
APC	antigen-presenting cell
Arf1	ADP-ribosylation factor 1
CU-3	5-[(2E)-3-(2-furyl)prop-2-enylidene]-3-[(phenylsulfonyl)amino]2-thioxo-1,3-thiazolidin-4-one)
DAG	diacylglycerol
DGK	diacylglycerol kinases
DGKα	the α paralog of human DGK
DGKε	the ε paralog of human DGK
ECM	extracellular matrix
ERK	extracellular signal-regulated kinase
FasL	Fas ligand
HIF1α	hypoxia-inducible factor 1-α
IL2	interleukin 2
IS	immunological synapse
Lck	lymphocyte-specific protein tyrosine kinase
LFA-1	lymphocyte function-associated antigen-1
MAPK	mitogen-activated protein kinase
MHC	major histocompatibility complex
MTOC	microtubule organizing center
mTOR	mammalian target of rapamycin
MVB	multivesicular bodies
NF-kB	nuclear factor kappa-light chain enhancer of activated B cells
NPC	nuclear pore complex
PA	phosphatidic acid
PC	phosphatidylcholine
PE	phosphatidylethanolamine
PI	phosphatidylinositol
PI-3,4-P_2_	phosphatidylinositol-3,4-bisphosphate
PI-3,4,5-P_3_	phosphatidylinositol-3,4,5-triphosphate
PI3K	phosphatidylinositol-3-kinase
PIP_2_	phosphatidylinositol-4,5-biphosphate
PIPn	phosphorylated forms of phosphatidylinositol
PKC	protein kinase C
PKD	protein kinase D
PLC	phospholipase C
PM	plasma membrane
PS	phosphatidylserine
SMAC	supramolecular activation cluster
Rab11-FIP1	Rab11 family of interacting protein 1
Rac1	Ras-related C3 botulinum toxin substrate 1
RasGAP	Ras GTPase activating protein
RasGRP1	a Ras guanyl nucleotide-releasing protein
RCP	Rab-coupling protein
Src	proto-oncogene tyrosine protein kinase
TCR	T-cell receptor
TGN	trans-Golgi network
TNFα	tumor necrosis factor α
